# Geriatrische Syndrome mit intensivmedizinischer Relevanz

**DOI:** 10.1007/s44179-022-00093-z

**Published:** 2022-10-21

**Authors:** Stephan Schmid, Sophie Schlosser, Martina Müller-Schilling

**Affiliations:** Klinik und Poliklinik für Innere Medizin I, Gastroenterologie, Hepatologie, Endokrinologie, Rheumatologie und Infektiologie, Universitätsklinikum Regensburg, Regensburg, Deutschland

Auf den Intensivstationen in Europa werden immer mehr ältere Patient*innen behandelt. Bis zu 30 % der insgesamt auf Intensivstation versorgten Patient*innen sind 80 oder mehr Jahre alt [1]. Gründe hierfür sind die demografische Entwicklung, die personalisierte Medizin, die medizinische und technische Weiterentwicklung in der Intensivmedizin sowie die COVID-19-Pandemie [2, 3]. Deshalb ist es auch im Kontext der COVID-19-Pandemie sehr wichtig zu berücksichtigen, dass das kalendarische Alter der Patient*innen allein kein prognostisches Kriterium für das Outcome einer intensivmedizinischen Versorgung ist [4]. Dies ist auch ein zentraler Punkt im Konsensuspapier zur geriatrischen Intensivmedizin der Deutschen Gesellschaft für Internistische Intensivmedizin und Notfallmedizin (DGIIN), der Österreichischen Gesellschaft für Internistische und Allgemeine Intensivmedizin und Notfallmedizin (ÖGIAIN), der Deutschen Interdisziplinären Vereinigung für Intensiv- und Notfallmedizin (DIVI), der Deutschen Gesellschaft für Geriatrie (DGG), der Österreichische Gesellschaft für Geriatrie & Gerontologie (ÖGGG), und weiterer Fachgesellschaften [5].

## Funktioneller Status

**Figure 1:**
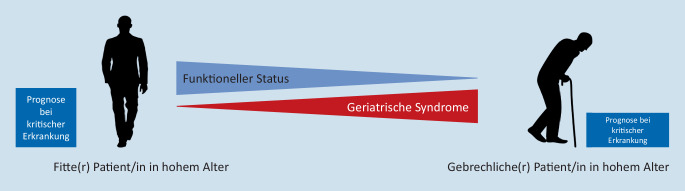

Viel wichtiger als das kalendarische Alter ist für die intensivmedizinische Prognose der *funktionelle Status* der Patient*innen vor der aktuellen Hospitalisation. Dieser wird von potenziell vorhandenen *geriatrischen Syndromen *mitbestimmt. Hierbei geht in der Gesamtbevölkerung ein hohes kalendarisches Alter statistisch mit einem reduzierten funktionellen Status einher; bei der/dem individuellen Patientin/Patienten bedingt ein hohes Alter nicht zwingend einen reduzierten funktionellen Status [5]. Abb. 1 zeigt den Zusammenhang zwischen funktionellem Status, geriatrischen Syndromen und der Prognose bei einer kritischen Erkrankung.

## Geriatrische Syndrome

Unter geriatrischen Syndromen versteht man klinische Zustände bei alten Patient*innen, die sich nicht in klassische Krankheitskategorien einordnen lassen. Sie sind wichtige Prädiktoren für das intensivmedizinische Outcome [6]. Dabei spielen insbesondere Frailty und Delir eine wichtige Rolle. In Tab. 1 wird eine Übersicht dieser geriatrischen Syndrome einschließlich Prävalenz und Definition und dargestellt.

**Table 1:** 

	Prävalenz	Definition
*Frailty* (altersbedingte Gebrechlichkeit)	Bis zu 50 % der älteren Menschen [7]	Erhöhte Vulnerabilität mit gesteigertem Risiko für ungünstiges intensivmedizinisches Outcome [8]
*Delir*	Bis zu 83 % der Patient*innen auf der Intensivstation [9]	Unspezifisches, polymorphes, hirnorganisches Syndrom, welches nicht allein durch Intoxikation mit Alkohol oder psychotropen Substanzen verursacht wird [10]

## Frailty

Frailty ist als klinischer Zustand definiert, welcher die Anfälligkeit von älteren Menschen für Stressoren erhöht [10]. Bis zu 50 % der Patient*innen im Alter von 80 oder mehr Jahren gelten als frail. In der Gesamtbevölkerung geht ein hohes Alter mit einer Zunahme der Frailty einher, das hohe Alter ist aber nicht allein ausschlaggebend für eine Zunahme der Frailty. Viele verschiedene Faktoren wie genetische Prädisposition oder Umwelteinflüsse spielen eine wichtige Rolle [7]. Gebrechliche Patient*innen haben bei einer intensivmedizinischen Behandlung ein deutlich erhöhtes Morbiditäts- und Mortalitätsrisiko, weiterhin kommt es nach einer intensivmedizinischen Behandlung signifikant häufiger zu funktionellen Beeinträchtigungen, oft mit Notwendigkeit der Aufnahme ins Pflegeheim [8].

**Figure 2:**
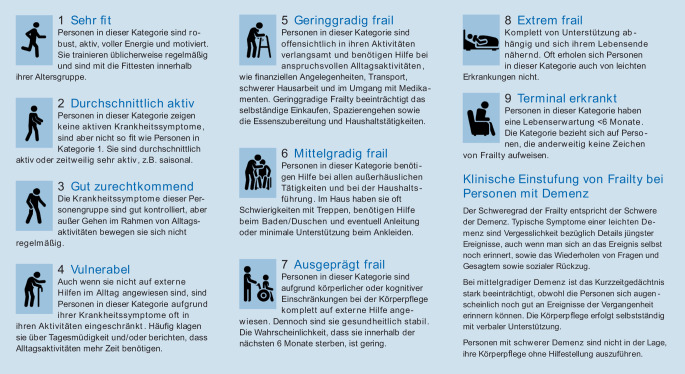

Die Einschätzung der Frailty ist für die intensivmedizinische Therapiestrategie von besonderer Bedeutung. Eine geeignete Methode hierfür auf der Intensivstation ist die Clinical Frailty Scale (CFS) bzw. klinische Frailty-Skala [12]. Die CFS ist in einer deutschen Übersetzung und den entsprechenden Piktogrammen auf der Homepage der deutschen Gesellschaft für Geriatrie (DGG) e. V. unter https://www.dggeriatrie.de/images/Bilder/PosterDownload/200331_DGG_Plakat_A2_Clinical_Frailty_Scale_CFS.pdf verfügbar und in Abb. 2 dargestellt [11]. Wichtig bei der Erhebung der Clinical Frailty Scale ist, dass von den Patient*innen, Angehörigen, Pflegenden, etc. erfragt wird, welchen Zustand die betreffende Person zwei Wochen vor der jeweiligen akuten Situation hatte. Auf keinen Fall darf sich also die Einschätzung auf den aktuellen Ist-Zustand, in der sich die betreffende Person befindet, beziehen.

Verschiedene europäische Studien konnten bei der intensivmedizinischen Behandlung von Patient*innen mit höherem Lebensalter, welche an Covid-19 erkrankt waren, die Bedeutung von Frailty zur Abschätzung der Mortalität und des Verlaufs zeigen [12–16]. Ein hohes Alter der Patient*innen allein ist ungeeignet, um den intensivmedizinischen Verlauf vorherzusagen oder die Morbidität und Mortalität abzuschätzen.

Die Gebrechlichkeit ist also auch in der COVID-19-Pandemie ein wichtiger Prädiktor für die Prognose von geriatrischen Patient*innen auf der Intensivstation und gut mithilfe der Clinical Frailty Scale (CFS) einschätzbar.

## Delir

Neben Frailty spielt das Delir eine sehr wichtige Rolle bei der Behandlung von älteren Patient*innen auf der Intensivstation. Das Delir ist die häufigste psychiatrische Erkrankung auf der Intensivstation. Bei beatmeten Patient*innen ist die Inzidenz sehr hoch und beträgt bis zu 83 % [17].

Per Definition handelt es sich beim Delir um ein ätiologisch unspezifisches, polymorphes, hirnorganisches Syndrom, welches nicht allein durch Intoxikation mit Alkohol oder psychotrope Substanzen verursacht wird und mindestens zwei der folgenden Störungen des Bewusstseins aufweist: Störungen der Aufmerksamkeit, der Wahrnehmung, des Denkens, des Gedächtnisses, der Psychomotorik, der Emotionalität oder des Schlaf‑/Wachrhythmus [18].

Die Bandbreite der Symptome eines Delirs ist sehr unterschiedlich. Drei Formen des Delirs können anhand der Symptomausprägung klassifiziert werden: hypoaktive Form, hyperaktive Form und gemischte Form [19]. Diesen Subtypen kann eine unterschiedliche Prognose zugeordnet werden: Patient*innen mit hypoaktivem Delir haben die schlechteste Überlebensprognose, da das Delir oft spät erkannt wird [20].

Bei der Entwicklung des Delirs spielen Risikofaktoren eine besondere Bedeutung. Hierbei unterscheidet man prädisponierende Risikofaktoren, präzipitierende Risikofaktoren und mit der medizinischen Versorgung assoziierte Risikofaktoren. Bei prädisponierenden Risikofaktoren handelt es sich z. B. um Gebrechlichkeit, Komorbiditäten oder Alkoholmissbrauch. Präzipitierende Risikofaktoren umfassen z. B. die akute medizinische Behandlung, Sepsis, Hypoglykämie oder Medikamente. Zusätzlich zu diesen prädisponierenden und präzipitierenden Faktoren spielen mit der medizinischen Versorgung assoziierte Risikofaktoren, wie z. B. mechanische Beatmung oder therapeutische Eingriffe, eine wichtige Rolle [17].

Die Leitlinien empfehlen zur Diagnose des Delirs die Confusion Assessment Method for Intensiv Care Unit (CAM-ICU) oder die Intensiv Care Delirium Screening Checklist (ICDSC) [8]. Hierbei hat die CAM-ICU-Methode eine Sensitivität von 80,0 % und eine Spezifität von 95,9 % und gilt deshalb als beste Screeningmethode für ein Delir auf der Intensivstation [19]. Das Delir ist mit einem sowohl kurz- als auch langfristig schlechten Outcome assoziiert. Die Mortalität von Patient*innen mit Delir ist 2‑ bis 3‑fach erhöht [20].

Zusammenfassend handelt es sich beim Delir um die häufigste psychiatrische Erkrankung auf der Intensivstation. Das Delir ist mit einem schlechten Outcome assoziiert. Eine frühzeitige Diagnosestellung mit Einleitung einer adäquaten Therapie ist daher essenziell für das Überleben der Patient*innen auf der Intensivstation.

## Multimorbidität und Polypharmazie

Neben den oben dargestellten geriatrischen Syndromen Gebrechlichkeit und Delir spielen für die intensivmedizinische Versorgung in Anlehnung an das aktuelle Statement der American Heart Association (AHA) Multimorbidität und Polypharmazie eine wichtige Rolle [3, 5].

Multimorbidität gilt als ein zunehmendes Phänomen im hohen Lebensalter. Da es keine einheitliche Definition gibt, ist die Prävalenz schwierig zu bestimmen. Diese liegt bei älteren Menschen zwischen 55 und 98 % [21]. Der Zusammenhang zwischen Multimorbidität und Frailty ist noch nicht ausreichend geklärt. Hierbei handelt es sich um zwei grundlegend verschiedene Konzepte. Gebrechlichkeit kann auch ohne Multimorbidität nachweisbar sein [22, 23].

Neben Multimorbidität ist bei der Versorgung von geriatrischen Patient*innen auf der Intensivstation die Polypharmazie von großer Bedeutung. Hierunter versteht man die dauerhafte Einnahme von fünf oder mehr Medikamenten. Multimorbidität ist nicht zwangsläufig mit Polypharmazie assoziiert, gilt jedoch als stärkster Prädiktor für Polypharmazie [24]. Deshalb ist bei der intensivmedizinischen Versorgung von geriatrischen Patient*innen eine enge Zusammenarbeit mit der Krankenhausapotheke von besonderer Bedeutung.

## Ernährungssituation der geriatrischen Intensivpatient*innen

Neben den geriatrischen Syndromen sowie Multimorbidität und Polypharmazie spielt die Ernährungssituation bei geriatrischen Intensivpatient*innen eine besonders wichtige Rolle. Viele geriatrische Krankenhauspatient*innen weisen bereits zum Zeitpunkt der Krankenhausaufnahme eine Mangelernährung auf, bei anderen besteht ein hohes Risiko für die Entwicklung einer solchen. Ziel ist es, den Ernährungszustand nicht nur zu erhalten, sondern die Situation zu verbessern und eventuelle Defizite zu beseitigen [5, 25–27]. Ein vorbestehendes Ernährungsdefizit geht mit einer erhöhten intrahospitalen Mortalität einher [28].

**Figure 3:**
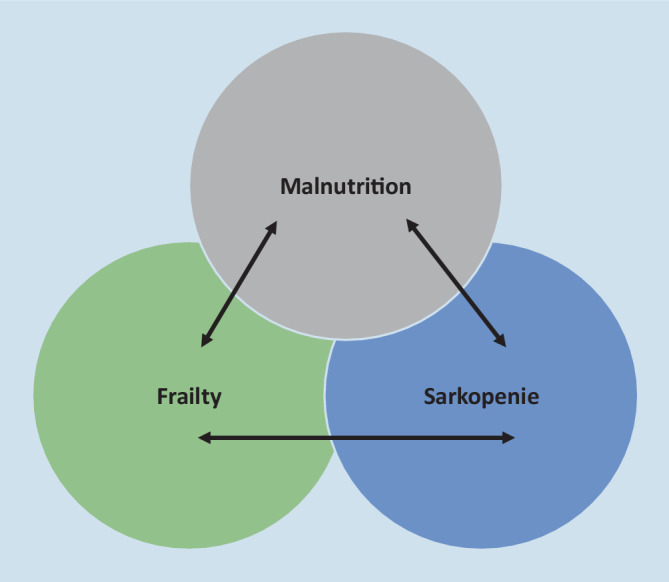

Eine Malnutrition kann ein wesentlicher Faktor für das Auftreten von Frailty und Sarkopenie sein. Sarkopenie muss nicht zwingend mit einer Mangelernährung einhergehen. Es besteht eine Wechselwirkung zwischen einer funktionellen Beeinträchtigung im Rahmen von Frailty und dem Ernährungsstatus in beide Richtungen. Malnutrition kann ein wesentlicher Faktor für das Auftreten von Frailty sein [29]. Das komplexe Zusammenspiel zwischen Malnutrition, Frailty und Sarkopenie wird in Abb. 3 dargestellt.

Unter Mangelernährung bei Intensivpatient*innen versteht man den Mangel an Makronährstoffen im Sinne einer Proteinenergiemalnutrition sowie den Mangel an Mikronährstoffen. Die Proteinenergiemalnutrition tritt bei 40 % der älteren Krankenhauspatient*innen auf [30]. Ein Screening hierbei ist z. B. mithilfe des Nutritional Risk Score (NRS) 2002 zur Abschätzung des Risikos einer Mangelernährung möglich [31, 32]. Eine umfassende Validierung von ernährungsmedizinischen Scores für den intensivmedizinischen Bereich ist ausstehend [26].

Eine Mangelernährung bei jüngeren Patient*innen ist meist auf eine dezidierte Ursache zurückzuführen. Bei älteren Patient*innen ist eine Malnutrition jedoch in der Regel ein multifaktorielles Geschehen, z. B. auf Dysphagie, Demenz oder krankheitsassoziierte Inappetenz zurückzuführen. Die Folgen einer Proteinenergiemalnutrition bei älteren Patient*innen sind gravierend und tragen unter anderem zu muskulärem Abbau sowie zur Verschlechterung der Wundheilung bei [33].

Weiterhin ist bei geriatrischen Patient*innen stets die Gefahr des Refeeding-Syndroms zu beachten. Hierbei gilt es besonders kritische Elektrolyte zu monitoren. Eine Dysphagie muss schnell erkannt und behandelt werden, um eine Aspiration zu verhindern [5].

## Therapiealgorithmus für geriatrische Patient*innen auf der Intensivstation

**Figure 4:**
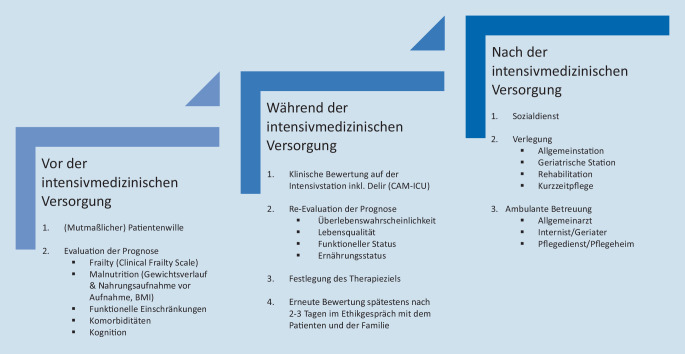

Basierend auf diesen Überlegungen zu den geriatrischen Syndromen sowie Multimorbidität, Polypharmazie und der Ernährungssituation wurde in unserer Klinik und Poliklinik für Innere Medizin I am Universitätsklinikum Regensburg ein Therapiealgorithmus zur intensivmedizinischen Versorgung für kritisch kranke geriatrische Patient*innen entwickelt (Abb. 4; [34]). Dieser umfasst die Versorgung von geriatrischen Patient*innen auf der Intensivstation und zugleich die Versorgung in der Notaufnahme sowie die Weiterbehandlung im stationären sowie ambulanten Bereich.

Bei der Versorgung von geriatrischen Patient*innen in der Notaufnahme ist der mutmaßliche Patientenwille von zentraler Bedeutung. Können Patient*innen ihren Willen nicht mehr selbst äußern, muss dieser mit Hilfe der Angehörigen oder einer evtl. vorliegenden Patientenverfügung evaluiert werden.

Während der intensivmedizinischen Behandlung von geriatrischen Patient*innen ist eine ständige klinische Bewertung und Re-Evaluation der Prognose mit Festlegung der Therapieziele notwendig. Spätestens nach zwei bis drei Tagen der intensivmedizinischen Versorgung sollte eine erneute Bewertung der Situation erfolgen. Zukünftig kann die Eingruppierung der Patient*innen in reproduzierbare Phänotypen, die auf akutmedizinischen und geriatrischen Merkmalen beruhen, zur Prognoseeinschätzung und spezifischen intensivmedizinischen Therapie hilfreich sein [35].

Bei der Behandlung von geriatrischen Patient*innen ist es sehr wichtig, frühzeitig die Versorgung nach dem intensivmedizinischen Aufenthalt sowohl im ambulanten als auch stationären Setting zu klären [36]. Hierzu ist eine frühzeitige Einbindung von Allgemeinmediziner*innen, Geriater*innen und Internist*innen sowie des Sozial- und Pflegedienstes notwendig. Die sektorenübergreifende Zusammenarbeit hat eine herausragende Bedeutung bei der Behandlung geriatrischer Patient*innen auf der Intensivstation.

## Zusammenfassung

Eine intensivmedizinische Versorgung ist auch für kritisch kranke geriatrische Patient*innen sinnvoll. Hierbei ist das kalendarische Alter allein kein prognostisches Kriterium für das Outcome der intensivmedizinischen Versorgung. Vielmehr ist der funktionelle Status, der von den geriatrischen Syndromen maßgeblich mitbestimmt wird, von zentraler Bedeutung. Die wichtigsten geriatrischen Syndrome sind Frailty und Delir. Zudem müssen Multimorbidität und Polypharmazie und die Ernährungssituation beachtet werden. Die sektorenübergreifende Zusammenarbeit ist bei der Behandlung von geriatrischen Patient*innen auf der Intensivstation von sehr großer Bedeutung.
